# Supermicrosurgical Technique for Distal Digital Amputation Replantation in Children Under 3 Years Old: A Single-Center Retrospective Study

**DOI:** 10.3390/jcm15145694

**Published:** 2026-07-20

**Authors:** Shi Gao, Haiqiong Chen, Guoqiang Zhao

**Affiliations:** Department of Traumatology, Children’s Hospital, Zhejiang University School of Medicine, National Clinical Research Center for Children and Adolescents’ Health and Diseases, Hangzhou 310052, China; 6516021@zju.edu.cn (S.G.);

**Keywords:** children, distal digital replantation, vascular crisis, supermicrosurgery

## Abstract

**Objective:** Vascular anastomosis is technically challenging in young children due to their slender and delicate vessels, which causes the clinical treatment of distal digital amputation to be relatively difficult. This study aims to investigate the therapeutic efficacy of the supermicrosurgical technique in the replantation of distal digital amputation in children under 3 years old. **Methods:** A retrospective analysis was conducted on 13 children (13 digits) with distal digital amputation from January 2023 to January 2026. All children underwent distal digital replantation using the supermicrosurgical technique. Fingertip bloodletting therapy was applied depending on the availability of anastomotic veins. Postoperatively, all children received symptomatic and supportive treatments, including anticoagulation, anti-infection, and antispasmodic therapy. Follow-up evaluations included finger growth, appearance, nail growth, and sensory and motor function. Parental satisfaction was assessed using the Michigan Hand Outcomes Questionnaire (MHQ). **Results:** There were nine males and four females, aged 8.7–36 months. Causes of injuries included six cases of door crush, two of heavy object crush and five of machine-related trauma. The warm ischemia time of the amputated digits was 4.6–11.8 h (mean time: 6.8 h). According to the Modified Ishikawa Classification, there were four Type I, four Type II, two Type III, and three Type IV injuries. All children received successful replantation surgery using the supermicrosurgical technique and were followed up for 6 to 12 months. Complete survival of the replanted digits was achieved in 11 cases. Two cases developed necrosis secondary to arterial crisis, giving an overall complete survival rate of 84.6%. The survived replanted digits exhibited almost normal appearance, good vascular perfusion, intact nail beds, restored sensation, and satisfactory flexion and extension. The necrotic digits showed pulp atrophy and hook nail deformity. According to the MHQ, all parents were satisfied with the clinical outcomes. **Conclusions:** Distal digital amputation, especially fingertip amputation, is commonly encountered in children under 3 years old, and replantation should be performed. High-quality anastomosis of tiny blood vessels under the supermicrosurgical technique, combined with rigorous postoperative management, contributes to a high survival rate and better clinical outcomes in children under 3 years old with distal digital replantation.

## 1. Introduction

Children’s inherent liveliness, curiosity, and hyperactivity, combined with limited life experience and insufficient self-protection awareness, make pediatric digit amputation a relatively common clinical injury. Most injuries involve distal digital amputation, including fingertip amputation [1,2]. Common causes include door entrapment, crush injuries from heavy objects, and mechanical trauma. Following pediatric digit amputation, parents typically have strong expectations for replantation. However, management is challenging due to the extreme delicacy of pediatric vessels and nerves, as well as difficulties in postoperative care. The distal digital vessels in pediatric patients are extremely thin with delicate walls, and it is difficult to expose lumens, all of which pose substantial challenges to intraoperative manipulation [3,4]. This is particularly the case for distal digital amputation in children under 3 years old, which requires not only advanced microsurgical skills but also meticulous perioperative management. With advances in microsurgical instruments, equipment, and sutures, supermicrosurgery has emerged as an extension of conventional microsurgery [5]. This technique enables high-quality anastomosis of the tiny blood vessels in young children. Currently, its application in pediatric distal digital replantation has significantly improved survival, while effectively restoring appearance and function [6]. Nonetheless, distal digital replantation in this group remains particularly challenging, and dedicated reports remain scarce in the literature. This study presents the surgical methods and clinical outcomes of supermicrosurgical replantation for distal digital amputation in children under 3 years old treated in our hospital.

## 2. Materials and Methods

### 2.1. Patient Population

This retrospective study analyzed 13 cases (13 digits) of traumatic distal digital amputation in children under 3 years old, from January 2023 to January 2026, who were treated in the Department of Traumatology, Children’s Hospital of Zhejiang University School of Medicine. Inclusion criteria were: (1) under 3 years old; (2) isolated traumatic distal digital amputation; and (3) follow-up duration exceeding 6 months with complete clinical data. Exclusion criteria included: (1) severe tissue damage of the amputated digit precluding replantation; (2) family refusal of replantation surgery; and (3) loss to follow-up. Informed consent was obtained from all children’s parents.

### 2.2. Clinical Classification and Prognostic Assessment

The Modified Ishikawa Classification [7] was used to classify the distal digital amputation. Finger growth, appearance, nail growth, sensory and motor function were selected as observation indicators. Parental satisfaction was assessed using the Michigan Hand Outcomes Questionnaire (MHQ) [8,9].

### 2.3. Surgical Procedures

Patients were anesthetized with a brachial plexus block combined with general anesthesia and placed in the supine position. An inflatable tourniquet was applied above the elbow. Under the operating microscope, the injured fingers were debrided to remove fine contaminants and contused tissues; the amputated digital segments were then debrided, followed by Kirschner wire fixation based on phalangeal fracture status. No phalangeal bone was resected for shortening, and soft tissue fixation was performed for phalangeal-free segments. For Modified Ishikawa Classification Type I and II injuries, a mid-volar longitudinal incision was made on the finger pulp, but for Type III and IV injuries, a volar lateral incision was designed according to the course of the digital arteries. Arteries and nerves were identified and marked. After digital reapproximation, tension-free skin closure was ensured, with supplementary sutures placed laterally away from neurovascular bundles for stabilization. Damaged tendons were repaired if indicated. The vascular diameters of all children were approximately 0.15–0.3 mm. Under ≥25× microscopic magnification, the injured digital arteries were trimmed and routinely irrigated with heparin sodium solution. After confirming vessel lumen integrity, end-to-end anastomosis was performed with 4–6 stitches using 11–0 polyamide monofilament nylon sutures, which were first placed at the 6 and 12 o’clock positions. Great caution was taken during needle insertion to avoid piercing the opposite vascular wall. Suture traction was performed gently to minimize trauma to the vessel wall. The tourniquet was released to verify arterial patency, followed by epineurial end-to-end neurorrhaphy for digital nerves. After confirming distal finger reperfusion (manifested by a pink, viable appearance), an attempt was made to identify suitable volar or dorsal digital veins for anastomosis. Finally, the wound was closed with interrupted tension-free sutures to complete the operation.

### 2.4. Postoperative Management

Postoperatively, the affected limb was immobilized with an above-elbow plaster cast in a flexed position and suspended on the chest. Strict bed rest was maintained, and measures were taken to minimize crying in children. Pharmacological management included anticoagulation, anti-infection, and antispasmodic therapy [10,11]. Fluid replacement was adjusted according to the children’s body weight and dietary intake. For children with impaired venous return, the distal digital bloodletting incision was intermittently swabbed with 50 U/mL heparin sodium solution. Initially, the incision was wiped every 30 min, but the interval could be extended if oozing was excessive. In case of venous crisis, the incision was inspected for thrombosis, and any thrombus was curetted if present. Over time, incision oozing gradually decreased until it ceased completely, with the finger pulp becoming reddish and well-perfused. For children without Kirschner wire fixation, the plaster cast was removed at 2 weeks postoperatively, and rehabilitation was initiated after complete wound healing. For those with Kirschner wire fixation, both the wires and cast were removed at 4 weeks, based on radiographic evidence of periosteal reaction observed, followed by targeted functional rehabilitation.

## 3. Results

**Figure 1:**
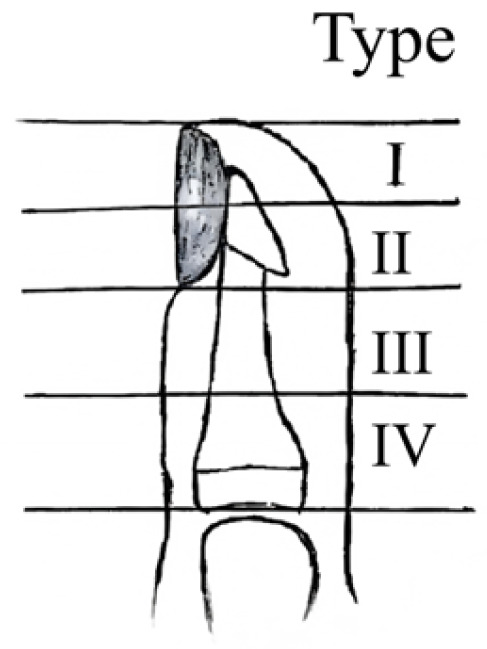
The modified Ishikawa classification of distal digital amputation. (Type I: Amputation distal to the distal half of the nail bed. Type II: Amputation distal to the nail root fold. Type III: Amputation from the nail root fold to the distal half of the distal interphalangeal joint. Type IV: Amputation from the distal interphalangeal joint to the proximal half of the nail root fold).

There were nine males and four females, aged 8.7–36 months. Causes of injuries included six cases of door crush, two of heavy object crush and five of machine-related trauma. The warm ischemia time of the amputated digits ranged from 4.6 to 11.8 h (mean: 6.6 h). According to the Modified Ishikawa Classification (Figure 1), there were four cases of Type I, four of Type II, two of Type III and three of Type IV categories. Types I and II are defined as fingertip amputations, including eight cases (Table 1).

**Figure 2:**
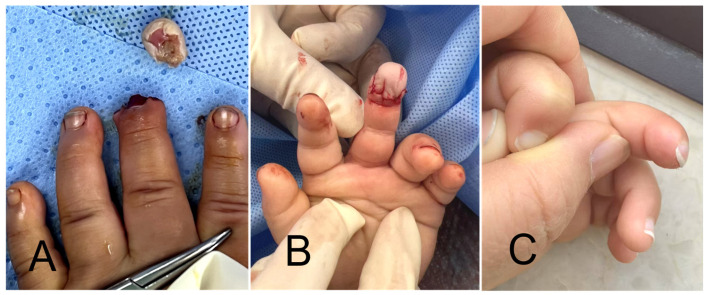
The preoperative, intraoperative, and postoperative periods of a child with a necrotic digit. (**A**) A 17-month-old male with a door-crush injury, resulting in complete amputation of the distal left middle digit beyond the nailbed. (**B**) The replanted digit presented a full contour with a reddish appearance. (**C**) Digital necrosis occurred, with a hook nail deformity developing at 11 months postoperatively.

**Figure 3:**
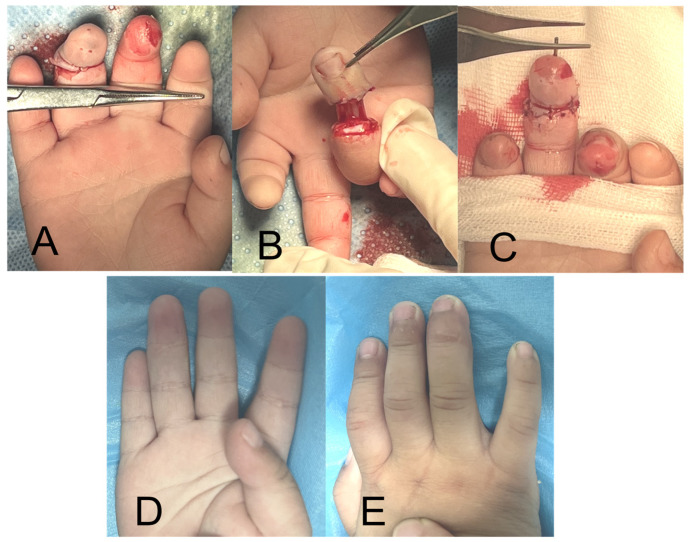
The preoperative, intraoperative, and postoperative periods of a child with a successfully replanted surviving digit. (**A**,**B**) An 8.7-month-old male with machine-related trauma involving both the palmar and dorsal surfaces. Only the deep flexor tendon remained connected, and no bleeding was observed after needling the fingertip. (**C**) Intraoperative appearance of the replanted right ring digit, showing restored blood supply with a pink, well-perfused pulp. (**D**,**E**): Palmar and dorsal appearances of the replanted digit at 10 months after survival.

The supermicrosurgical technique was applied in all replantation procedures, with arterial and nerve anastomosis performed in all cases. In this cohort, 10 cases of Type I, II and III injuries had no suitable veins, so a dermal incision was made at the distal tip for bloodletting to prevent venous crisis. In contrast, three cases of Type IV injuries underwent successful dorsal digital vein anastomosis. All children were followed up for 6–12 months. Among the 13 cases, 11 achieved complete survival of the replanted digits, with an overall success rate of 84.6% (Table 1). Two patients who underwent Type I amputation developed necrosis secondary to arterial crisis. Although the wound healed with dressing changes, the necrotic digits exhibited a flattened pulp contour and hook nail deformity (Figure 2). The survived replanted digits exhibited almost normal appearance, good vascular perfusion, intact nail beds, restored sensation, and satisfactory flexion and extension (Figure 3). Parental satisfaction was assessed using the MHQ, and all parents were satisfied with the treatment outcomes.

## 4. Discussion

Through this study, we conclude that the successful survival of distal digital replantation in children under 3 years old is associated with multiple factors, such as the Modified Ishikawa Classification, the quality of vascular anastomosis using the supermicrosurgical technique, and postoperative management.

### 4.1. Is There a Correlation Between Modified Ishikawa Classification and Distal Digit Replantation Survival?

The commonly used scale for distal digit amputation is the Modified Ishikawa Classification, which mainly includes four types. This classification is relatively refined, with different types corresponding to distinct amputation planes [7,12]. Types I/II are generally referred to as fingertip amputations. Most cases in children under 3 years old involve fingertip amputation, further complicating replantation. The distal digital arteries and nerves in children are extremely thin, with a diameter usually less than 0.3 mm, and they become even thinner in fingertip amputation injuries [13,14,15]. The thinner the blood vessel, the more difficult it is to perform suturing, the higher the requirements for suture quality, and the more likely a vascular crisis will occur, resulting in necrosis of the replanted finger [16]. The two necrotic cases were classified as Type I. This type presents the most distal amputation plane with the thinnest vessels. Therefore, whether survival outcomes are obtained after distal digital replantation may be correlated with the Modified Ishikawa Classification, and further research is needed to confirm the correlation between them.

### 4.2. How Can the Quality of Vascular Anastomosis Be Improved?

Vascular anastomosis quality is the key to the survival of replanted digits. It is difficult for conventional microsurgical techniques to accomplish anastomosis of tiny blood vessels. However, the advent of the supermicrosurgical technique makes anastomosis of tiny blood vessels feasible [17]. At the 1st European Congress of Super-Microsurgery in 2010, the Barcelona Consensus defined supermicrosurgery as a technique for microvascular and neural anastomosis and dissection of small-caliber vessels or single-nerve fascicles, with the applicable vessel diameter specified as 0.3–0.8 mm [18]. With the rapid advancement of supermicrosurgery, the patency rate of small-vessel anastomosis has been greatly improved, showing clear technical advantages and promoting its widespread use worldwide [19]. Using high-magnification surgical microscopes, supermicrosurgery provides a clearer operative view and enables faster and more accurate identification of tiny vessels. With delicate microsurgical instruments and sutures, vessels once considered unsuitable for anastomosis can now be successfully reconstructed. Currently, the technique is widely applied for anastomosis of vessels thinner than 0.3 mm, significantly enhancing anastomotic quality [6,20]. Meanwhile, the precise manipulation afforded by supermicrosurgery reduces iatrogenic injury to skin and soft tissues during dissection. With the application of the supermicrosurgical technique, high-quality tiny vascular anastomosis can be accomplished by means of interrupted everting sutures, which maintain proper stitch intervals and prevent injury to the contralateral vascular wall. In the present series, all 13 cases underwent replantation using the supermicrosurgical technique. High-quality vascular anastomosis ensured patency of the anastomotic stomas, and successful replantation surgery was obtained in every case.

### 4.3. Postoperative, Individualized Management Is Needed to Reduce the Occurrence of Vascular Crisis

Postoperative management plays an essential role in the prevention of vascular crisis. Vascular crisis is the most dangerous complication after finger replantation, so relevant measures should be taken postoperatively to prevent its occurrence [21,22]. In addition to routine interventions—including environmental smoke-free conditions, warming, antispasmodics (Papaverine: 1.5 mg/kg, administered every 8 h for one week), anticoagulation (Dextran 40 and glucose: 5 mL/kg, administered every 12 h for one week), and antibiotics—effective communication with parents is crucial to prevent vascular crisis [23]. It remains our persistent goal to implement targeted treatment based on pediatric characteristics, avoid vascular crises, tide over the critical stage smoothly, and guarantee the survival of replanted digits. In the present series of 13 patients, 10 received postoperative bloodletting therapy. We observed that approximately 5 days were required for re-establishment in children undergoing fingertip bloodletting, and none of the children received a blood transfusion. The delayed venous suture technique has been widely applied in adult vascular repair [24,25]. However, its implementation in pediatric patients remains technically challenging and difficult to perform in clinical practice. Shorter recovery time was noted in younger children and those with milder tissue injuries, which was consistent with previous reports in the literature [26,27]. Therefore, dressing changes should be minimized within the first 5 postoperative days, and efforts should be made to keep the children calm, which can help reduce vascular crisis caused by crying. Furthermore, parents should be informed of the treatment protocol and instructed to monitor the color of the replanted digits, facilitating clinical care and timely detection of vascular crisis. The measures we advised parents to perform included taking a photograph of the replanted digits and providing timely feedback to the medical team. These postoperative strategies effectively stabilized the children’s emotions, reduced the use of sedative medications, and relieved parental anxiety. We consider that such measures are of great importance in reducing the incidence of vascular crisis and improving the survival of replanted digits.

Finally, this study has several limitations. Firstly, it was a single-center retrospective study with a small sample size and heterogeneous injury mechanisms and amputation levels. Secondly, the follow-up period was relatively short. Only short-term efficacy and safety were evaluated, whereas long-term outcomes—including finger growth and development and their impact on psychological development in patients—were not fully investigated. Finally, no comparison with conventional microsurgical techniques was made. Further studies with larger samples, longer follow-up periods, and multicenter controlled designs are therefore warranted.

## 5. Conclusions

Distal digital amputation in children under 3 years old should be treated with replantation whenever feasible. High-quality anastomosis of tiny blood vessels under the supermicrosurgical technique, together with rigorous postoperative management, contributes to a high survival rate and satisfactory clinical outcomes. It offers a certain referential value for the management of children with similar conditions.

## Figures and Tables

**Table 1:** Clinical data of 13 cases of distal digital amputation.

Number	Age (Months)	Gender	Amputated Digit	Injury Pattern	Modified Ishikawa Classification	Warm Ischemic Time (Hour)	Vascular Anastomosis (Artery/Vein)	Replantation Outcomes
1	33	female	right index digit	machine-related	IV	6.2	1/1	survival
2	23	male	right index digit	heavy object crush	III	6.8	1/0	survival
3	17	male	left middle digit	door crush	I	7.3	1/0	necrosis
4	30	female	right index digit	machine-related	III	11.8	1/0	survival
5	21	female	right middle digit	machine-related	I	5.8	1/0	survival
6	27	male	right ring digit	door crush	II	5.4	1/0	survival
7	24	male	left middle digit	door crush	II	6.1	1/0	survival
8	16	male	right middle digit	door crush	II	6.4	1/0	survival
9	36	female	right index digit	machine-related	IV	4.6	1/1	survival
10	8.7	male	right ring digit	machine-related	IV	7.4	1/1	survival
11	36	female	right middle digit	door crush	I	8.2	1/0	necrosis
12	32	male	right ring digit	heavy object crush	II	5.7	1/0	survival
13	27	male	left middle digit	door crush	I	6.5	1/0	survival

## Data Availability

The original contributions presented in this study are included in the article. Further inquiries can be directed to the corresponding authors.
